# Supplementary material for: Detection of ferromagnetic resonance from 1 nm-thick Co
Source: Sci Rep. 2020 Sep 25;10:15764. doi: 10.1038/s41598-020-72760-7 (PMC7519674; doi:10.1038/s41598-020-72760-7)
Supplement: Supplementary file 1 — Supplementary Information. [file 41598_2020_72760_MOESM1_ESM.docx]

**Supplementary Information**

**Detection of Ferromagnetic Resonance from 1 nm-thick Co**

Shugo Yoshii, Ryo Ohshima, Yuichiro Ando, Teruya Shinjo and Masashi Shiraishi ^†^

Department of Electronic Science and Engineering, Kyoto University, Nishikyo-ku,

Kyoto 615-8510, Japan

1. **FMR spectrum from the Co (1 nm)/Ta (1 nm) sample.**

Fig. S1 shows the FMR spectrum from the Co (1 nm)/Ta (1 nm) sample. In contrast to the results of the other Co/Ta samples, no FMR signal can be seen. This directly shows that the Ta buffer layer of 1 nm in thickness does not assist formation of 1-nm-thick Co, where FMR can be excited.


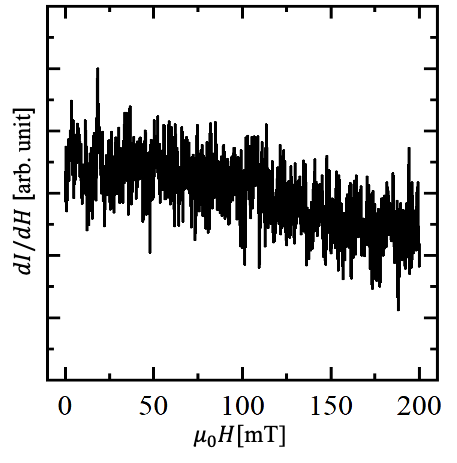


Fig. S1 FMR spectra the Co (1 nm)/Ta (1 nm) sample.

1. **Deconvolution of the FMR spectra.**

To determine the Gilbert damping constant *α*, deconvolution of the FMR spectra was carried out using the integral form of the spectra. Figure S2 shows the integral form of the FMR spectra of Co (1 nm)/Ta (*d* nm, *d*=2,3,4 and 5 nm) samples. The zero of the horizontal axis is set to be the resonance field of each sample. The result of the deconvolution for the Co (1 nm)/Ta (3 nm) sample using the fitting function described in the main text is shown in Fig. S3. Although non-negligible asymmetric component is superposed in the spectrum, the magnitude of the asymmetric component is sufficiently small and the symmetric component, i.e. the Lorentzian component, is dominant. The resonance field, the half-width at half-maximum and the *α* were estimated from the result of the fitting.


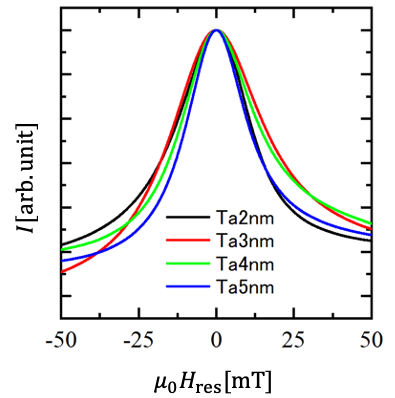


Fig. S2 Integral form of the FMR spectra from the Co (1 nm)/Ta (*d* nm, *d*=2,3,4 and 5 nm) samples.


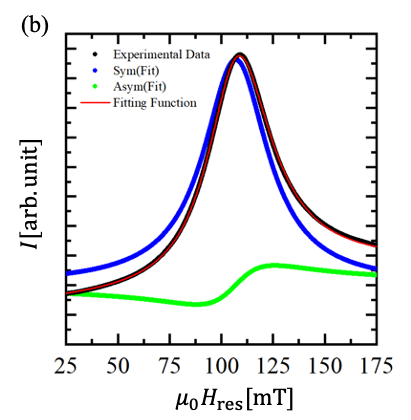


Fig. S3 Result of the deconvolution of the FMR spectrum of the Co (1 nm)/ Ta (3 nm) sample.
